# Impact of Cardiovascular Magnetic Resonance Imaging on Identifying the Etiology of Cardiomyopathy in Patients Undergoing Cardiac Transplantation

**DOI:** 10.1038/s41598-018-34648-5

**Published:** 2018-11-01

**Authors:** Lucy Q. Lin, Felipe Kazmirczak, Ko-Hsuan Amy Chen, Osama Okasha, Prabhjot S. Nijjar, Cindy M. Martin, Mehmet Akçakaya, Afshin Farzaneh-Far, Chetan Shenoy

**Affiliations:** 1 Department of Medicine, University of Minnesota Medical Center, Minneapolis, Minnesota USA; 2 Cardiovascular Division, Department of Medicine, University of Minnesota Medical Center, Minneapolis, Minnesota USA; 3 Department of Electrical and Computer Engineering and Center for Magnetic Resonance Research, University of Minnesota, Minneapolis, Minnesota USA; 4 Division of Cardiology, Department of Medicine, University of Illinois at Chicago, Chicago, Illinois USA

## Abstract

Errors in identifying the etiology of cardiomyopathy have been described in patients undergoing cardiac transplantation. There are increasing data that cardiovascular magnetic resonance imaging (CMR) provides unique diagnostic information in heart failure. We investigated the association of the performance of CMR prior to cardiac transplantation with rates of errors in identifying the etiology of cardiomyopathy. We compared pre-transplantation clinical diagnoses with post-transplantation pathology diagnoses obtained from the explanted native hearts. Among 338 patients, there were 23 (7%) errors in identifying the etiology of cardiomyopathy. Of these, 22 (96%) occurred in patients with pre-transplantation clinical diagnoses of non-ischemic cardiomyopathy (NICM). Only 61/338 (18%) had CMRs prior to transplantation. There was no significant association between the performance of CMR and errors in the entire study cohort (p = 0.093). Among patients with pre-transplantation clinical diagnoses of NICM, there was a significant inverse association between the performance of CMR and errors (2.4% vs. 14.6% in patients with and without CMR respectively; p = 0.030). In conclusion, CMR was underutilized prior to cardiac transplantation. In patients with pre-transplantation clinical diagnoses of NICM – in whom 96% of errors in identifying the etiology of cardiomyopathy occurred – the performance of CMR was associated with significantly fewer errors.

## Introduction

Heart failure is associated with significant mortality, morbidity, and healthcare costs. An important prognostic factor in heart failure is the etiology of the underlying cardiomyopathy^1^. Etiology-specific treatment, in addition to standard heart failure therapies, can slow disease progression, reverse myocardial remodeling, and delay or preclude the need for advanced therapies such as cardiac transplantation^2^. Examples of such etiology-specific therapies include: coronary revascularization for ischemic cardiomyopathy (ICM), immunosuppression for inflammatory cardiomyopathies such as cardiac sarcoidosis and giant cell myocarditis, and exercise restriction and implantable cardioverter defibrillators for arrhythmogenic cardiomyopathy^2^. Thus, accurate identification of the etiology of cardiomyopathy may result in improved heart failure outcomes.

Errors in identifying the etiology of cardiomyopathy have been described in patients undergoing cardiac transplantation through comparisons of pre-transplantation clinical diagnoses with post-transplantation diagnoses obtained by pathology examination of the explanted native hearts^3–7^. The error rates were 8–21%, with the majority of errors involving non-ischemic cardiomyopathies (NICM)^3–7^.

Cardiovascular magnetic resonance imaging (CMR) has an important role in the evaluation of cardiomyopathy^8,9^. Tissue characterization of the myocardium using the late gadolinium enhancement technique (LGE CMR) frequently helps identify the etiology of a cardiomyopathy^10–13^. Whether CMR could help lower the rates of errors in identifying the etiology of cardiomyopathy is unknown. To investigate the impact of CMR, we studied consecutive patients that underwent cardiac transplantation and examined whether the performance of CMR any time prior to transplantation (hereon noted as pre-transplantation CMR) was associated with fewer errors in identifying the etiology of cardiomyopathy.

## Methods

### Overview

We performed a retrospective, observational study using the University of Minnesota’s Cardiovascular Magnetic Resonance Registry^14^ and the Cardiac Transplantation Registry. We hypothesized that performance of pre-transplantation CMR would be associated with fewer errors. The study was approved by University of Minnesota’s Institutional Review Board with a waiver of informed consent. It was performed in accordance with relevant guidelines and regulations.

### Study cohort

Consecutive adult patients who underwent cardiac transplantation at the University of Minnesota from January 1, 2004 through December 31, 2017 were included. This time period was selected to match that of ready clinical availability of CMR at our institution.

### Methods

CMRs were performed on a Siemens 1.5T scanner as per standardized protocols^15,16^ and included cine CMR for the assessment of left and right ventricular structure and function, and tissue characterization of the myocardium using the late gadolinium enhancement technique (LGE CMR).

Demographic and clinical data, and pre- and post-transplantation cardiomyopathy diagnoses were independently extracted from patient records by two investigators (L.Q.L. and C.S.). Differences between the investigators were resolved by consensus. Data collected on pre-transplantation diagnostic testing included the performance of: echocardiography, coronary angiography, endomyocardial biopsy, pathology evaluation of the apex of the left ventricle (LV) obtained at the time of left ventricular assist device (LVAD) implantation, and CMR.

Since patients are often referred to our institution for cardiac transplantation, referral records were also reviewed to identify diagnostic testing performed outside of our institution prior to cardiac transplantation. Five (1.5%) patients with CMRs did not have LGE CMR and were excluded from the final analysis.

Pre-transplantation diagnoses were defined as those made by the cardiac transplantation team using clinical, hemodynamic and diagnostic testing data, and documented in the patients’ records prior to transplantation. Post-transplantation diagnoses were defined as those made by pathologists on routine pathology analyses of explanted hearts. ICM by pathology was recognized by the presence of ischemic-pattern (transmural or subendocardial, in a coronary artery distribution) replacement fibrosis, and the absence of findings suggestive of a non-ischemic etiology (inflammation, granulomas, etc.).

As in previous studies^3–7^, errors were defined as discordances between pre-transplantation clinical diagnoses and post-transplantation pathology diagnoses. For patients with NICM diagnoses, the specific etiology of NICM was compared.

### Statistics

Statistical analyses were performed using Stata 13 (StataCorp LP, College Station, Texas, USA). Parametric continuous variables were expressed as means with standard deviation (SD). Categorical variables were expressed as counts with percentages. Comparison between groups was performed with a 2-sample Student t test for continuous, normal variables, and Wilcoxon rank sum test for continuous, non-normal data. Categorical data were compared using either chi-squared test or Fisher’s exact test. All tests were two-tailed, and p < 0.05 was considered statistically significant.

## Results

During the study period, 341 patients underwent cardiac transplantation. Of these, three (0.9%) patients were excluded because their pathology reports for explanted hearts were not available. The pre-transplantation clinical diagnoses for the excluded patients were: idiopathic dilated cardiomyopathy, idiopathic restrictive cardiomyopathy and complex congenital heart disease. The remaining 338 patients comprised the study cohort.

### Errors in identifying the etiology of cardiomyopathy

Details of pre- and post-transplantation cardiomyopathy diagnoses are provided in Table 1. Of 338 study patients, 152 (45%) had pre-transplantation clinical diagnoses of ICM. Overall, there were errors in identifying the etiology of cardiomyopathy in 23 (7%) study patients. Details of individual patients with errors are listed in Table 2.

**Table 1:** Pre- and Post-Transplantation Cardiomyopathy Diagnoses.

Etiology of cardiomyopathy	Number of patients with pre-transplantation clinical diagnosis	Number of patients with post-transplantation pathology diagnosis
ICM	152	158
NICM	186	180
Idiopathic or familial dilated cardiomyopathy	102	90
Complex congenital heart disease	16	16
Hypertrophic cardiomyopathy	12	14
Valve disease-associated cardiomyopathy	12	10
Anthracycline-associated cardiomyopathy	10	9
Cardiac sarcoidosis	7	6
Left ventricular non-compaction cardiomyopathy	5	5
Arrhythmogenic cardiomyopathy (includes ARVC and LDAC)	5	8
Restrictive cardiomyopathy (idiopathic or radiation-related)	4	4
Alcoholic cardiomyopathy	3	2
Peripartum cardiomyopathy	2	2
Neuromuscular cardiomyopathy	2	2
Myocarditis	1	4
Cardiac amyloidosis	1	3
Chagas cardiomyopathy	1	1
Neutral storage lipid disease	1	1
Transplant allograft vasculopathy	1	1
Chronic allograft rejection	1	1
Giant cell myocarditis	0	1

ARVC = arrhythmogenic right ventricular cardiomyopathy; ICM = ischemic cardiomyopathy; LDAC = left dominant arrhythmogenic cardiomyopathy; NICM = non-ischemic cardiomyopathy.

**Table 2:** Individual Patients with Errors in Identifying the Etiology of Cardiomyopathy.

Patient Number	Pre-transplantation clinical diagnosis	Post-transplantation pathology diagnosis	Pre-transplantation echocardiography	Pre-transplantation coronary angiography	Pre-transplantation endomyocardial biopsy	Pre-transplantation LVAD	Pre-transplantation CMR
1	Alcoholic cardiomyopathy	Ischemic cardiomyopathy	Yes	Yes	No	Yes	No
2	Anthracycline-associated cardiomyopathy	Giant cell myocarditis	Yes	Yes	No	No	No
3	Cardiac sarcoidosis	Idiopathic dilated cardiomyopathy	Yes	Yes	No	No	No
4	Cardiac sarcoidosis	Idiopathic dilated cardiomyopathy	Yes	Yes	Yes	No	No
5	Familial dilated cardiomyopathy	Hypertrophic cardiomyopathy	Yes	Yes	No	Yes	No
6	Idiopathic dilated cardiomyopathy	LV non-compaction cardiomyopathy	Yes	Yes	No	No	No
7	Idiopathic dilated cardiomyopathy	Myocarditis	Yes	No	No	Yes	No
8	Idiopathic dilated cardiomyopathy	Cardiac amyloidosis	Yes	No	No	No	No
9	Idiopathic dilated cardiomyopathy	Hypertrophic cardiomyopathy	Yes	Yes	Yes	Yes	No
10	Idiopathic dilated cardiomyopathy	Ischemic cardiomyopathy	Yes	Yes	No	No	No
11	Idiopathic dilated cardiomyopathy	Ischemic cardiomyopathy	Yes	Yes	No	Yes	No
12	Idiopathic dilated cardiomyopathy	Cardiac amyloidosis	Yes	Yes	No	Yes	No
13	Idiopathic dilated cardiomyopathy	Ischemic cardiomyopathy	Yes	Yes	No	Yes	No
14	Idiopathic dilated cardiomyopathy	Myocarditis	Yes	Yes	No	Yes	No
15	Idiopathic dilated cardiomyopathy	Ischemic cardiomyopathy	Yes	Yes	No	Yes	No
16	Idiopathic dilated cardiomyopathy	Ischemic cardiomyopathy	Yes	Yes	No	No	No
17	Idiopathic dilated cardiomyopathy	Arrhythmogenic cardiomyopathy	Yes	Yes	Yes	Yes	No
18	Idiopathic dilated cardiomyopathy	Ischemic cardiomyopathy	Yes	No	No	Yes	No
19	Idiopathic dilated cardiomyopathy	Arrhythmogenic cardiomyopathy	Yes	Yes	No	Yes	No
20	Ischemic cardiomyopathy	Idiopathic dilated cardiomyopathy	Yes	Yes	No	Yes	No
21	LV non-compaction cardiomyopathy	Arrhythmogenic cardiomyopathy	Yes	Yes	No	Yes	Yes
22	Valvular cardiomyopathy	Cardiac sarcoidosis	Yes	Yes	No	No	No
23	Valvular cardiomyopathy	Myocarditis	Yes	Yes	No	No	No

CMR = cardiovascular magnetic resonance imaging; LV = left ventricular; LVAD = left ventricular assist device.

Among patients with pre-transplantation clinical diagnoses of ICM, one (0.5%) had a post-transplantation diagnosis of idiopathic dilated cardiomyopathy. Among the 186 (55%) patients with pre-transplantation clinical diagnoses of NICM, there were errors in identifying the etiology of cardiomyopathy in 22 (12%). Thus, 22/23 (96%) of all errors occurred in patients with clinical diagnoses of NICM.

Among patients with errors after clinical diagnoses of NICM, ICM was the most common missed diagnosis, accounting for 30% (7/23) of errors. NICM diagnoses that were missed include: three cases each of myocarditis, idiopathic dilated cardiomyopathy and arrhythmogenic cardiomyopathy, two cases each of cardiac amyloidosis and hypertrophic cardiomyopathy, and one case each of cardiac sarcoidosis, giant cell myocarditis and left ventricular non-compaction cardiomyopathy.

### Comparison of patients with and without pre-transplantation CMRs

Patient characteristics stratified by performance of pre-transplantation CMR are listed in Table 3. CMR was performed pre-transplantation in 61 (18%) patients, of which, 19 (31%) were performed in patients with pre-transplantation clinical diagnoses of ICM. Patients with pre-transplantation CMR were younger, more likely to be female, and more likely to have had an endomyocardial biopsy.

**Table 3:** Comparison of patients with and without pre-transplantation CMRs.

	Patients with pre-transplantation CMR (n = 61)	Patients without pre-transplantation CMR (n = 277)	p value
Age at transplantation, years	47.3 ± 15.5	55.4 ± 11.7	<**0**.**01**
Male sex, n (%)	37 (60.7)	211 (76.2)	**0**.**013**
Pre-transplantation echocardiography, n (%)	61 (100.0)	277 (100.0)	1.000
Pre-transplantation coronary angiography, n (%)	47 (77.0)	213 (76.9)	0.979
Pre-transplantation endomyocardial biopsy, n (%)	14 (23.0)	20 (7.2)	**0**.**0002**
Pre-transplantation LVAD, n (%)	29 (47.6)	163 (59)	0.107
Errors in identifying the diagnosis of cardiomyopathy in the entire cohort (n = 338)	1 (1.6)	22 (7.9)	0.093
Errors in identifying the diagnosis of cardiomyopathy in patients with clinical diagnoses of ICM (n = 152)	0/19 (0.0)	1/133 (0.8)	1.000
Errors in identifying the diagnosis of cardiomyopathy in patients with clinical diagnoses of NICM (n = 186)	1/42 (2.4)	21/144 (14.6)	**0**.**030**

CMR = cardiovascular magnetic resonance imaging; ICM = ischemic cardiomyopathy; LVAD = left ventricular assist device; NICM = non-ischemic cardiomyopathy.

### Associations of errors with the performance of pre-transplantation CMRs

There was no significant association between the performance of pre-transplantation CMR and errors overall – 1.6% in patients with pre-transplantation CMR vs. 7.9% in those without transplantation CMR; p = 0.093. Among patients with pre-transplantation clinical diagnoses of ICM, there was no association between the performance of pre-transplantation CMR and errors. Among patients with pre-transplantation clinical diagnoses of NICM, there was a significant inverse association between the performance of pre-transplantation CMR and errors – 2.4% in patients with pre-transplantation CMR vs. 14.6% in those without transplantation CMR; p = 0.030. Thus, patients with pre-transplantation clinical diagnoses of NICM and pre-transplantation CMRs had 86% lower odds of having an error in identifying the etiology of cardiomyopathy compared with those with pre-transplantation clinical diagnoses of NICM and no pre-transplantation CMRs.

## Discussion

In a contemporary comparison of pre-transplantation clinical diagnoses of cardiomyopathy with post-transplantation pathology diagnoses, there were errors in 7% of the study cohort. While the performance of CMR pre-transplantation was not associated with fewer errors overall, it was associated with significantly fewer errors in the subgroup with pre-transplantation clinical diagnoses of NICM. The latter accounted for 96% of all errors.

Coronary angiography is conventionally used to identify ICM versus NICM. However, this approach is limited because the absence of obstructive coronary artery disease (CAD) does not always rule out ICM; in a recent systematic review of myocardial infarction with non-obstructive coronary arteries, 24% of patients had late gadolinium enhancement (LGE) in an ischemic pattern, possibly due to spontaneous healing of acute thrombotic occlusion from atherosclerotic CAD or thromboembolic disorders, or coronary vasospasm^17^. This may explain errors in the six patients with pre-transplantation diagnoses of NICM after coronary angiography, but with post-transplantation diagnoses of ICM. Conversely, the presence of obstructive CAD in a patient with cardiomyopathy does not always signify ICM and may simply be incidental. This may explain the error in the patient with pre-transplantation clinical diagnosis of ICM. Through identification of the ischemic pattern of LGE characterized by involvement of the subendocardium (i.e. subendocardial or transmural) and location in the perfusion territory of an epicardial coronary artery, CMR can accurately distinguish between ICM and NICM^12,18^.

Although multiple studies have previously demonstrated the role of CMR for the identification of the etiology of cardiomyopathy, they have all defined ICM based on the presence of significant CAD by coronary angiography^10,19–21^. Additionally, all these studies investigated the role of CMR in distinguishing ICM from NICM, and none have systematically investigated its role in identifying the specific etiology of NICM.

**Figure 1:**
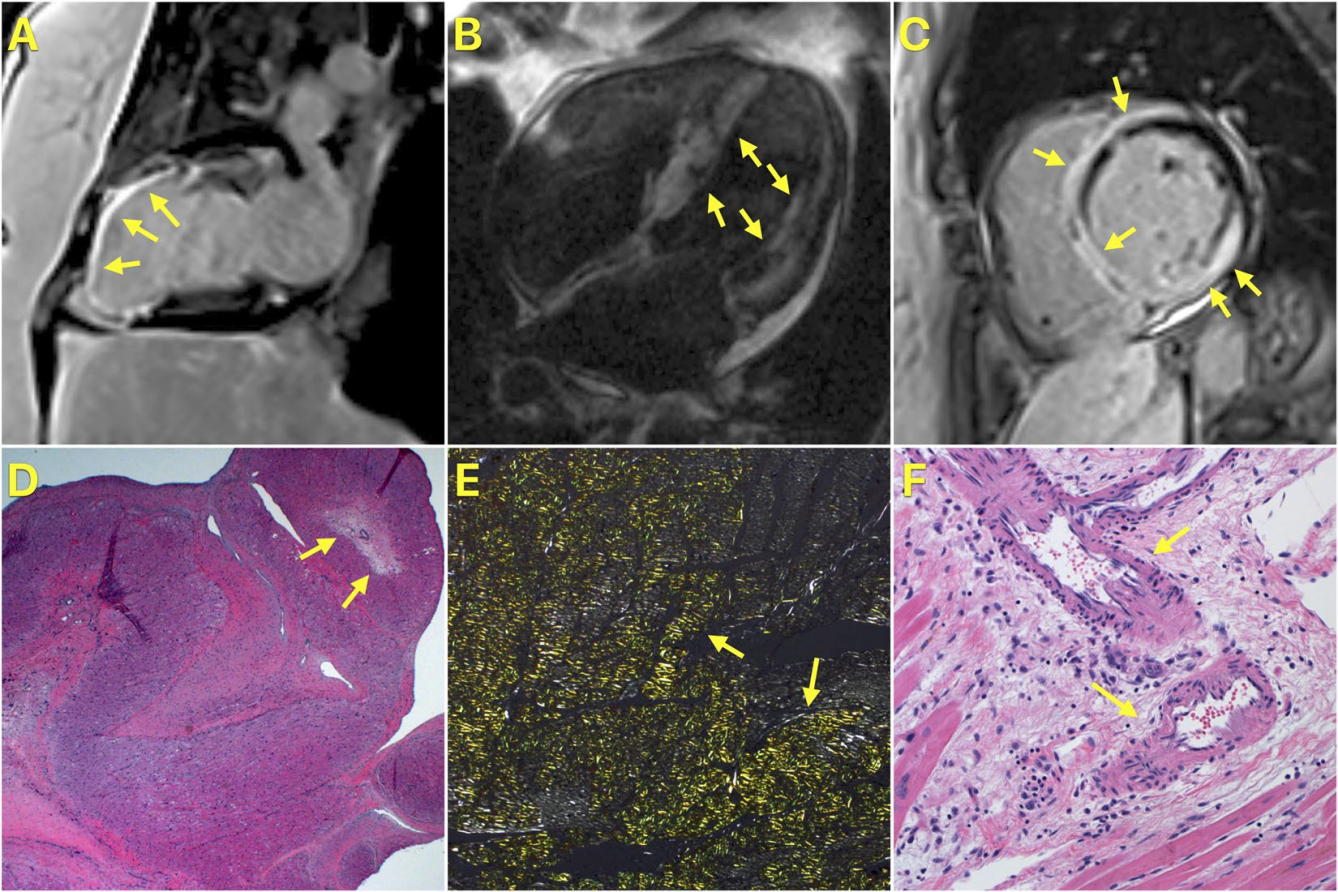
Examples of Study Patients with CMRs and No Errors in Identifying the Etiology of Cardiomyopathy. Panel A is a two-chamber LGE CMR image of a patient with ischemic cardiomyopathy. Yellow arrows point to transmural LGE in the distribution of the left anterior descending coronary artery. Panel B is a four-chamber LGE CMR image of a patient with cardiac amyloidosis. Yellow arrows point to diffuse transmural LGE; also note the low signal intensity of the blood in the cardiac chambers, which is also typical for cardiac amyloidosis. Panel C demonstrates a basal short-axis LGE CMR image of a patient with cardiac sarcoidosis. Yellow arrows point to LGE involving multiple segments in epicardial, transmural and near-transmural patterns; also note involvement of the right side of the interventricular septum, which is typical for cardiac sarcoidosis. Panel D is a pathology image from the same patient as in Panel A showing subendocardial myocyte loss and replacement fibrosis (yellow arrows) consistent with a healed ischemic myocardial infarction (magnification 10x; hematoxylin and eosin stain). Panel E is a pathology image from the same patient as in Panel B showing apple-green birefringence of amyloid protein (yellow arrows) in polarized light (magnification 20x; Congo red stain). Panel F is a pathology image of the interventricular septum from the same patient as in Panel C showing foci of perivascular inflammation comprising predominantly of histiocytes and lymphocytes. Although this pattern of inflammation is non-specific, it is consistent with treated sarcoidosis. Also seen are foci of replacement fibrosis (magnification 50x; hematoxylin and eosin stain). Non-caseating granulomas were previously seen on endomyocardial biopsy performed after the CMR identified cardiac sarcoidosis.

Identification of the specific etiology of NICM by CMR relies on the recognition of specific LGE phenotypes based on locations and patterns^12^. Examples include diffuse transmural LGE for cardiac amyloidosis, and multifocal, epicardial LGE with involvement of the right ventricular aspect of the basal interventricular septum for cardiac sarcoidosis (Fig. 1). Absence of LGE narrows the differential diagnosis to idiopathic dilated cardiomyopathy, familial cardiomyopathy, stress cardiomyopathy, peripartum cardiomyopathy, and toxic (alcoholic or anthracycline-related) cardiomyopathy. Frequently, interpretation of a patient’s LGE phenotype in the context of other clinical data yields a specific etiology for the NICM. Newer techniques such as T1 mapping can also help in identifying etiologies of NICM such as cardiac amyloidosis and Fabry cardiomyopathy.

**Figure 2:**
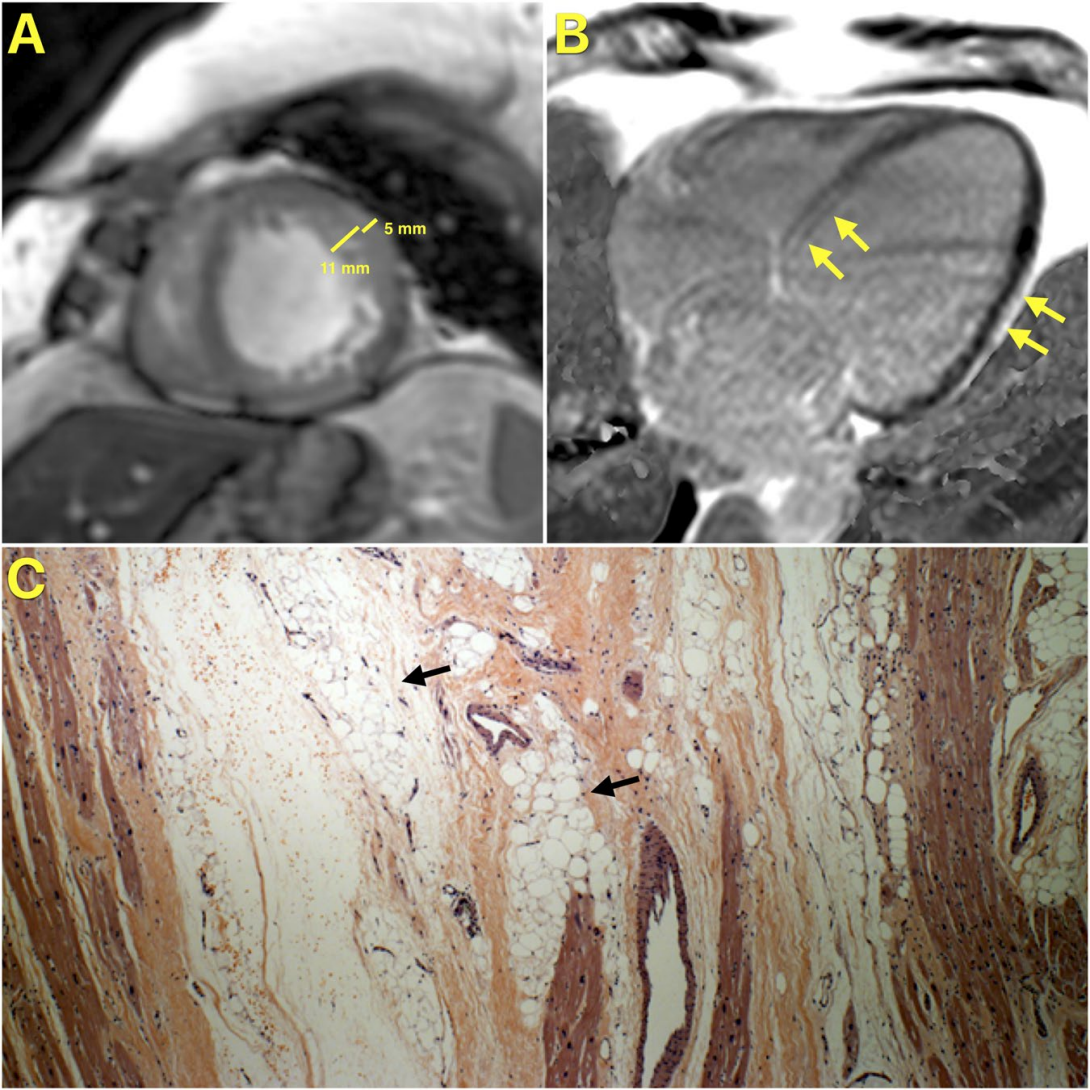
Study Patient with CMR and Error in Identifying the Etiology of Cardiomyopathy. Panel A is a short axis cine-CMR image showing excessive trabeculations with a noncompaction/compaction ratio at end-systole of 2.2. Panel B is a four-chamber LGE CMR image of the same patient showing epicardial LGE in the mid-lateral wall (yellow arrows) and mid-myocardial LGE in the basal septum (yellow arrows). Panel C shows a pathology image from the same patient demonstrating extensive fatty infiltration of the myocardium (black arrows).

The lone NICM case missed despite a pre-transplantation CMR had a pre-transplantation clinical diagnosis of left ventricular non-compaction cardiomyopathy and on post-transplantation pathology examination was found to have arrhythmogenic cardiomyopathy with biventricular involvement, related to a mutation in the LMNA gene (Fig. 2). The patient met criteria for the diagnosis of left ventricular non-compaction cardiomyopathy; the noncompaction/compaction ratio measured on a short axis image at end-systole was 2.2 (>2 denotes the presence of left ventricular non-compaction cardiomyopathy)^22^. Patients fulfilling criteria for left ventricular non-compaction cardiomyopathy have previously been described to have left-dominant arrhythmogenic cardiomyopathy^23^.

Our overall error rate is on the lower end of the range seen in prior studies of errors in identifying the etiology of cardiomyopathy in patients undergoing cardiac transplantation^3–7^ (Table 4). This may be due to the availability of CMR during our entire study period, and its use in 18%. Previous studies included patients that underwent cardiac transplantation prior to the pioneering publications of LGE CMR in 1999–2001^24–26^.

**Table 4:** Comparison of Current Study with Prior Studies Comparing Cardiomyopathy Pre-Transplantation Clinical Diagnoses with Post-Transplantation Pathology Diagnoses.

Study	Study period	Cardiac transplantation center	n	Study patients	Errors in identifying the diagnosis of cardiomyopathy
Bortman *et al*.^3^	06/1998–03/1993	University of Texas Southwestern Medical Center, Dallas, Texas, USA	112	All consecutive patients undergoing cardiac transplantation	16/112 (14%)
Angelini *et al*.^4^	11/1985–02/1994	University Medical School of Padua, Padua, Italy	257	All consecutive patients undergoing cardiac transplantation	20/257 (8%)
Luk *et al*.^5^	01/1987–07/2006	Toronto General Hospital, Toronto, Ontario, Canada	296	All consecutive patients undergoing cardiac transplantation	51/296 (17%)
Mehra *et al*.^6^	01/1992–08/2003	Ochsner Clinic Foundation, New Orleans, Louisiana, USA	112	Patients with pre-transplantation clinical diagnosis of non-ischemic cardiomyopathy*	23/112 (21%)
Roberts *et al*.^7^	03/1993–06/2012	Baylor University Medical Center, Dallas, Texas, USA	314	All consecutive patients undergoing cardiac transplantation	42/314 (13%)
Current study	01/2004–12/2017	University of Minnesota Medical Center, Minneapolis, Minnesota, USA	338	All consecutive patients undergoing cardiac transplantation^†^	23/338 (7%)

*Excluding age <35 years, congenital or familial cardiomyopathy, active myocarditis, peripartum cardiomyopathy, primary valvular heart disease or infiltrative cardiomyopathy.

^†^Excluding three patients with missing pathology reports.

While our study findings are based on patients with end-stage cardiomyopathy that subsequently underwent cardiac transplantation, they carry a greater implication for patients with newly-diagnosed cardiomyopathy. CMR was only performed in 18% of our study cohort, highlighting that it is greatly underutilized for the identification of the etiology of cardiomyopathy. Early and accurate diagnosis of the etiology of cardiomyopathy can guide etiology-specific treatment, with the potential for reversal of myocardial remodeling and recovery of systolic function, and avoidance of advanced treatments such as implantable cardioverter defibrillator therapy, cardiac resynchronization therapy, LVAD therapy and cardiac transplantation^2^.

## Limitations

Our study is limited by its retrospective, single-center design. Pre-transplantation diagnostic testing was performed at the discretion of the transplantation team. CMR was performed infrequently; many patients did not receive CMR because they were referred for cardiac transplantation from hospitals without CMR programs with implantable cardioverter-defibrillators in place, limiting the use of CMR. Newer CMR techniques such as T1 mapping were performed only in a recent minority of CMRs since the study period spanned 14 years. Patients with CMRs had a higher rate of endomyocardial biopsies; however, whether these were a consequence of CMR findings is not known. Post-transplantation pathology interpretations were not blinded to pre-transplantation clinical diagnoses. With regard to the implications, it may not be feasible to perform CMR in patients that present in a hemodynamically unstable state and require ventricular assist devices or cardiac transplantation before they are stable enough to undergo a CMR.

## Conclusions

Comparing pre-transplantation clinical diagnoses of cardiomyopathy with post-transplantation pathology diagnoses, there were errors in 7% of the study cohort. Ninety-six percent of all errors occurred in patients with pre-transplantation clinical diagnoses of NICM. In these patients, the performance of CMR pre-transplantation was associated with 86% lower odds of having an error. Prospective studies are needed to investigate whether routine use of CMR in patients with clinical diagnoses of NICM could improve clinical outcomes by decreasing errors in identifying the etiology of cardiomyopathy.
